# The Effect of VR Avatar Embodiment on Improving Attitudes and Closeness Toward Immigrants

**DOI:** 10.3389/fpsyg.2021.705574

**Published:** 2021-10-15

**Authors:** Vivian Hsueh Hua Chen, Gabrielle C. Ibasco, Vetra Jing Xuan Leow, Juline Yun Yee Lew

**Affiliations:** Wee Kim Wee School of Communication and Information, Nanyang Technological University, Singapore, Singapore

**Keywords:** avatar embodiment, virtual reality, empathy, social identity orientation, intergroup bias, anchoring

## Abstract

Past research has discussed how the embodiment of an outgroup avatar in virtual reality (VR) can reduce intergroup bias. However, little is known about the mechanisms and boundary conditions that shape this effect. This study examines how the embodiment of both outgroup and ingroup VR avatars in different orders influences attitudes and perceived closeness toward a co-ethnic immigrant outgroup in Singapore. It also investigates the role of empathy and social identity orientation (SIO) in this relationship. An experiment with four avatar embodiment conditions (ingroup-then-outgroup, outgroup-then-ingroup, ingroup-only, and outgroup-only) was carried out with 171 participants from a public university in Singapore. Results showed that embodying an outgroup avatar alone, compared to embodying an ingroup avatar alone, significantly improves both attitudes and closeness toward an immigrant outgroup. The order of embodiment matters to an extent, suggesting the greater effectiveness of outgroup-first over ingroup-first embodiment in reducing bias. Empathy mediates the effect of all three outgroup embodiment conditions on improved attitudes and closeness toward immigrants. It was also found that the stronger one’s SIO is, the more effective embodiment is in improving perceived closeness with the outgroup *via* empathy. Theoretical implications of these findings are discussed.

## Introduction

The last few years have precipitated a surge in media coverage of intergroup conflicts, including xenophobic online sentiments and hate crimes in the wake of COVID-19. Understanding and resolving tensions between the “ingroup” (i.e., group to which one belongs) and “outgroup” (i.e., group to which one does not belong) in various contexts has been a longstanding theoretical and practical endeavor. Extensive research has investigated how biases against outgroups can be reduced (see Dovidio et al., 2017; Paluck et al., 2021 for reviews). For example, seminal strategies such as intergroup contact have been found to improve attitudes toward outgroups by reducing anxiety about intergroup interactions (Pettigrew and Tropp, 2008). Other studies showed that perspective-taking can lead to stronger self-other overlap, or associative links between the self and an outgroup (Myers et al., 2014; Todd and Galinsky, 2014).

Apart from utilizing these traditional strategies, scholars have identified interactive technology such as virtual reality (VR) as novel, potential avenues for intergroup bias reduction. One distinct feature of VR is embodiment, which is defined as the sensation of “being inside, having, and controlling a [virtual] body” (Kilteni et al., 2012, p. 374). Through the affordances of visuo-motor synchrony and real-time action enabled by VR, embodiment allows a person to feel that they are controlling a virtual body as if it were their own (Tham et al., 2018). The embodied experience of inhabiting an avatar different from oneself can generate behavioral changes that align with that avatar (Yee and Bailenson, 2006), as well as changes in self-perception that suggest overlap between the avatar and self (Banakou et al., 2013, 2016).

Studies have explored how embodying participants in a virtual outgroup avatar can influence biases, especially toward a racial outgroup (Yee and Bailenson, 2006; Groom et al., 2009; Peck et al., 2013; Banakou et al., 2016; Behm-Morawitz et al., 2016b; Hasler et al., 2017; Salmanowitz, 2018; Breves, 2020). However, results have been inconclusive. Some have found that implicit attitudes (i.e., unconscious attitudes measured through response latencies toward valenced ingroup vs. outgroup associations) toward Black people improved through the virtual embodiment of a Black avatar (Peck et al., 2013; Banakou et al., 2016). Similar studies, however, found that embodiment of a Black avatar resulted in greater or no changes to implicit attitudes (Groom et al., 2009; Hasler et al., 2017).

Beyond methodological differences, one possible reason for these inconclusive results is that most studies have not empirically tested the underlying socio-cognitive mechanisms and boundary conditions shaping the effects of embodiment on intergroup bias. One study identified avatar liking as a moderator that enables the positive effects of embodiment in improving attitudes (Behm-Morawitz et al., 2016b). However, psychological variables central to the intergroup relations literature have yet to be investigated in relation to embodiment. Empathy, the process of understanding another’s emotions, has been found to be a key mechanism underlying the positive effects of multiple strategies, including intergroup contact (Pettigrew and Tropp, 2008) and perspective-taking (Vescio et al., 2003). At the same time, there may be potential obstacles to bias reduction, such as the cognitive tendency to anchor to an ingroup perspective (Hysenbelli et al., 2013), as well as the variability in latent predispositions to self-identify according to social groups (Brickson and Brewer, 2001).

Addressing multiple components of intergroup bias is also an ideal outcome for bias reduction research. A wealth of studies has shown that the aforementioned strategies can be effective in reducing affective (Tropp and Pettigrew, 2005) and cognitive dimensions (Myers et al., 2014) of intergroup bias. The present study focuses on both closeness (cognitive dimension) and affective attitudes (affective dimension) as primary outcomes. As a direct measure, attitudes refer to the valenced opinions or feelings one holds about a member of an outgroup (Wilcox et al., 1989). Closeness toward an outgroup is often measured as self-other overlap (Aron et al., 1992), which entails a blurring of group boundaries, precluding the propensity for ingroup favoritism and outgroup derogation (Galinsky and Moskowitz, 2000).

The present study tests the effect of VR embodiment on both attitudes and closeness toward an outgroup in a novel co-ethnic immigration context in Asia. To the best of our knowledge, no study has examined the effect of embodiment in a co-ethnic context, where virtual avatars are not differentiated by skin tone or major physiological identity markers. This study seeks to understand how embodiment of a Chinese foreign immigrant (outgroup) vs. a Chinese Singaporean citizen avatar (ingroup) influences Chinese Singaporeans’ attitudes toward immigrants. It examines the mechanisms and variables underlying how embodiment influences intergroup attitudes. Specifically, it aims to address how this effect is (1) shaped by ingroup anchoring effects (2) mediated by empathy, and (3) moderated by social identity orientation (SIO).

## Embodiment and Intergroup Bias

The current research predominantly supports the argument that outgroup embodiment can reduce negative intergroup biases (e.g., Yee and Bailenson, 2006; Peck et al., 2013; Banakou et al., 2016). In these studies, light-skinned or White ingroup participants in the manipulation condition are typically made to embody a dark-skinned or Black avatar, while the control group embodies their own ingroup (e.g., Peck et al., 2013; Banakou et al., 2016; Behm-Morawitz et al., 2016b). Peck et al. (2013) tested this manipulation with respect to implicit racial attitudes. In the VR environment, participants were simply instructed to move around, observe their VR avatar’s appearance, and test their synchronized movements. White participants embodied in a dark-skinned avatar experienced a greater reduction in implicit negative attitudes toward Black people than those embodied in a light-skinned avatar and those who perceived a non-embodied dark-skinned avatar.

Furthermore, Banakou et al. (2016) found that these positive effects of outgroup embodiment on implicit attitudes can be sustained long after the embodiment scenario. In one experiment, participants were embodied in a Black or White avatar and instructed to follow the movements of a Tai Chi instructor in a VR environment. They found that when compared to ingroup embodiment, embodiment of a Black outgroup avatar can significantly reduce implicit negative bias, even when measured one week after the embodiment manipulation.

However, one risk of outgroup representation in a mediated environment is stereotype activation. Stereotypes are cognitive “shortcuts” or mental representations that comprise automatic associations or assumptions made about a person based on their group identity. Stereotypes can be activated or made salient through situational cues in one’s environment, influencing perceptions and behavior (Wheeler and Petty, 2001). Such is the case in the study by Groom et al. (2009), where embodying White participants in a Black avatar resulted in increased implicit bias toward Black people. Unlike other studies, the VR environment used in this study modeled a job interview scenario, where Black people are often stereotyped to perform poorly (Peck et al., 2013), thus potentially activating latent negative stereotypes about Black people being subpar job candidates. Similarly, another study found that the stereotypical representation of a Black avatar can inadvertently increase negative judgments about the outgroup (Behm-Morawitz et al., 2016a).

Hasler et al. (2017) likewise found no significant difference in implicit attitude change between White participants who embodied a White ingroup avatar and those who embodied a Black avatar. Participants were instructed to interact with a programmed virtual partner, who was either Black or White and exhibited standardized behavior in a turn-taking task. Hasler et al. (2017) found through measuring avatar liking that participants varied greatly in their affinity toward this virtual partner. Greater liking of a Black virtual partner was significantly associated with lower implicit bias. As such, in this study, preferences for interaction partners may have confounded the relationship between avatar embodiment and implicit attitudes (Hasler et al., 2017).

Given the inconsistencies found in embodiment studies, the VR environment is designed to minimize the activation of negative stereotypes about the immigrant outgroup through a stereotype-neutral food court scenario. Brief interactions with multiple non-playable characters (NPCs; i.e., food court customers) and objects (i.e., food and drink ingredients) are included as part of the embodiment scenario, making it less likely for the impression of one character to confound overall attitudes. The NPCs are also designed to represent a diverse range of ethnic groups, so affinity toward these characters should not directly correlate with evaluations of the Chinese immigrant outgroup.

*H1*: Participants exhibit more positive changes in attitudes and perceived closeness toward an immigrant outgroup when embodied in an outgroup avatar than when embodied in an ingroup avatar.

To explain the outcomes of embodiment on intergroup outcomes, studies have frequently drawn parallels between embodiment and perspective-taking, the process of seeing things from another’s point of view (see Banakou et al., 2016; Herrera et al., 2018; Herrera, 2020). Perspective-taking has generally been found to strengthen associations between the “self” and “other,” promoting more positive intergroup evaluations or attitudes (Galinsky et al., 2005; Boca et al., 2018). However, one distinction is that embodiment is experiential while perspective-taking is imaginal. Perspective-taking manipulations typically require participants to imagine what another person may be going through. With embodiment, participants directly inhabit another’s experiences through body-ownership illusions (Banakou et al., 2016). Nonetheless, due to the two concepts’ shared theoretical associations with self-other overlap, we draw on research about the cognitive biases and mechanisms underlying perspective-taking to see how they may similarly apply to embodiment processes.

## Anchoring and Order Effects

Research suggests that attempts at perspective-taking may be hindered due to the anchoring heuristic (Keysar et al., 2000; Epley et al., 2004; Barr and Keysar, 2006). People rely on an initial piece of information (the “anchor”) to shape their understanding of subsequent stimuli, resulting in inaccurate attempts to deal with situations of ambiguity (Tversky and Kahneman, 1974; Doyle et al., 2020). One’s own egocentric or self-centered perspective may serve as the anchor to frame the understanding of another person’s viewpoint (Keysar et al., 2000).

People tend to anchor to opinions or judgments made by ingroup members over outgroup members (Hysenbelli et al., 2013; Hedgebeth, 2020). In one study, women were provided with written information about how much an exemplar donated to a charity, and were then asked how much they would be willing to donate themselves. This exemplar was either a member of their national ingroup or outgroup and reportedly donated either a high or low amount. A high anchor amount generated significantly greater donations when the anchor source was an ingroup member versus an outgroup member (Hysenbelli et al., 2013).

To avoid this anchoring bias, one may enact the adjustment process, which involves making sequential and deliberate changes to one’s initial perspective to account for new information about another’s viewpoint. However, adjustment may be limited due to the lack of a strong motivation for accuracy, the amount of cognitive resources required (Epley et al., 2004; Epley and Gilovich, 2006), as well as the accessibility or salience of anchor-consistent information (Mussweiler and Strack, 1999a,b). When subtle cues related to an ingroup identity were made initially salient, individuals exhibited greater subsequent ingroup loyalty and favoritism (Hertel and Kerr, 2001).

Apart from numerical estimation tasks, the anchoring bias has also been found to influence judgments about the affect and feelings others (Yik et al., 2019), as well as changes in attitudes and beliefs (Hogarth and Einhorn, 1992). In Hogarth and Einhorn’s (1992) model of belief adjustment, the process of adjusting one’s beliefs from an initial anchor may depend on the order in which a person is exposed to anchor-consistent or inconsistent evidence. For simple judgments (i.e., evaluating the likeability of a person based on a series of adjectives), where evaluation is made after all pieces of evidence are presented, greater adjustment from an anchor is made based on evidence presented earlier (i.e., primacy effect). In contrast, for complex judgments (i.e., evaluating arguments about cause-and-effect based on a series of event descriptions), stimuli presented later may drive adjustment (i.e., recency effect; Hogarth and Einhorn, 1992).

While anchoring-and-adjustment has been applied to perspective-taking, a similar logic may likewise inform embodiment, which can make salient different viewpoints through an immersive role-playing experience. The initial embodiment of a familiar ingroup character may reify an ingroup anchor, hindering the ability to embody a subsequent outgroup character. In line with Hogarth and Einhorn (1992), the order of ingroup vs. outgroup embodiment may affect how much weight people assign to an outgroup avatar’s viewpoint, subsequently influencing evaluative judgments about the outgroup.

To our knowledge, no research thus far has explored embodiment order effects when a person undergoes more than one embodiment experience sequentially. This study explores the role of both ingroup *and* outgroup embodiment consecutively. It tests whether the high accessibility of anchor-consistent information can influence the effect of virtual outgroup embodiment, which, compared to the imaginal methods of perspective-taking research, may be a more “robust” method of merging the self with the other (Herrera et al., 2018; Hasson et al., 2019). We counterbalanced the order of both embodiment scenarios to test for order effects. Based on the anchoring literature, we propose the following hypotheses:

*H2a*: Embodying the outgroup first (i.e., outgroup-only, outgroup-then-ingroup) results in a more positive change in attitudes and closeness toward the outgroup than embodying the ingroup first (i.e., ingroup-only, ingroup-then-outgroup).

*H2b*: When embodying the ingroup first, there are no significant differences in the change in attitudes and closeness induced by the single embodiment (i.e., ingroup-only) versus double embodiment conditions (i.e., ingroup-then-outgroup).

## Empathy

Empathy is an affective variable that has been studied in relation to both virtual embodiment and intergroup relations. It is defined as an emotional response to another’s feelings and experiences that may develop as a result of trait dispositions and situational cues (Cuff et al., 2016). Embodiment in VR, commonly discussed as an “empathy machine” (Herrera et al., 2018), can stimulate empathy by manipulating the user’s multi-sensory experiences (Bertrand et al., 2018) or by vividly conveying another’s emotions and experiences to the user (Shin, 2018).

Herrera et al. (2018) found that participants embodied in a homeless person in VR had greater empathy and longer-lasting positive attitudes toward the homeless than the control group. Empathy levels in the VR embodiment group were notably greater than those in the perspective-taking condition, where participants were simply asked to imagine the viewpoint of a homeless person.

Furthermore, studies on empathy outside of VR demonstrate how inducing empathy can improve attitudes toward outgroups (Batson et al., 1997; Shih et al., 2009). Across three experiments, Batson et al. (1997) found that high-empathy participants, who were instructed to imagine a stigmatized group member’s feelings, expressed more positive explicit attitudes toward the stigmatized group than did participants exposed to the low-empathy manipulation. Collectively, these findings give credence to a pathway model in which empathy results from embodiment and, in turn, may improve attitudes toward an outgroup.

Parallel to this body of work, empathy has also been identified as a mediator in the relationship between perspective-taking and intergroup attitudes in other research (Vescio et al., 2003; Shih et al., 2009). In one study, where participants were asked to imagine the perspective of the other, Vescio et al. (2003) found empathy to partially mediate the relationship between perspective taking and intergroup attitudes. Similar to, and possibly to a greater extent than perspective-taking, embodiment may nurture empathy by allowing people to viscerally integrate another’s viewpoint (Herrera et al., 2018), enhancing the sense that an avatar’s experiences are equivalent to one’s own. Given these findings, we hypothesize that:

*H3*: Empathy positively mediates the effect of outgroup embodiment on changes in attitudes and perceived closeness toward the outgroup.

## Social Identity Orientation

The outcomes of intergroup bias reduction can likewise be influenced by dispositional traits that shape how different individuals view the self in relation to others. According to the social categorization theory, individuals have latent predispositions to identify according to personal or social levels of the self (Brickson, 2000; Nario-Redmond et al., 2004). For example, personal identifiers may value their individuality and uniqueness, while social identifiers may focus on the importance of their belonging to particular groups (Nario-Redmond et al., 2004). Given the social nature of intergroup relations how they emerge from group identification, the differential importance people assign to their social selves may play a role in constructing biases and receptiveness to attitude change.

Studies have linked SIO with intergroup bias outcomes, but results have been somewhat conflicting. Research has shown that individuals who orient toward the personal level of the self may be more likely to leverage negative intergroup attitudes to distinguish themselves from others as unique individuals (Augoustinos and Walker, 1998). Conversely, those who gravitate toward the social level of the self may prioritize social equality and harmony, resulting in more favorable attitudes toward an outgroup (Gouveia, 2011). However, the opposite pattern may also be plausible—due to an emphasis on intergroup comparison and hierarchies, social identifiers may be less motivated to individuate or view others as individuals (Brickson and Brewer, 2001), and may instead rely on group-based stereotypes in their attitude formation (Perdue et al., 1990; Duclos and Barasch, 2014).

Additionally, studies have established links between SIO and empathy, our proposed mediator for the effect of embodiment on intergroup bias. People with a stronger awareness of their social group or identity are more likely to exhibit empathy (Preston and De Waal, 2002; Zhao et al., 2013) and are more attuned to the feelings and experiences of others (Cross et al., 2000). In a study by Duan et al. (2008), collectivism—the cultural value placed on the social collective—predicted greater emotional empathy. Likewise, in a neurological ERP study, priming a view of the self as socially interdependent on other people stimulated stronger neural empathic responses to the pain of others (Chen et al., 2020). Taken together, these studies suggest that a SIO may facilitate the prosocial effects of empathy, but this relationship has yet to be examined in the context of embodiment and intergroup bias. As research on the direct influence of SIO on intergroup bias also remains inconclusive, we propose a research question instead of a hypothesis:

*RQ3*: How does social identity orientation influence the indirect effect *via* empathy of outgroup embodiment in VR on attitudes and closeness toward the outgroup?

## The Research Context

This study was conducted in Singapore, an immigration heavy and multi-ethnic society in Southeast Asia. As of end June 2020, the city-state is home to a population of 5.69 million people (Department of Statistics Singapore, 2020) of which Chinese-ethnic Singaporeans comprise the majority (76%) of citizens (gov.sg, 2019).

Migrants (2.16 million) comprise close to 37% of the total population in Singapore (Hirschmann, 2020). Among the migrant groups, approximately 18% are Chinese migrants originating from the People’s Republic of China (PRC; United Nations, 2015). However, there is a notable ingroup-outgroup divide between the Singaporean Chinese majority (ingroup) and the PRC Chinese minority (outgroup). A few studies show that Singaporean Chinese hold negative views about PRC Chinese (Liu, 2014; Ortiga, 2015; Ramsay and Pang, 2017; Ahmed et al., 2021). Compared to other immigrant groups, PRC Chinese are stereotyped to be the least warm and most threatening to Singaporeans’ cultural values and economic resources (Authors, in Press; Ramsay and Pang, 2017). Such evidence of “co-ethnic prejudice” may stem from Singaporeans’ prioritization of national over racial identity in their views toward the PRC Chinese diaspora. Despite shared ethnic and cultural origins, PRC Chinese may be viewed as a novel outgroup based on perceived social and cultural differences, threats to scarce economic resources, and a lack of political loyalty to the Singaporean national identity (Liu, 2014).

This study examines the unique intergroup relations between co-ethnic Singaporean Chinese (ingroup) and PRC Chinese (outgroup). Few studies have examined embodiment in relation to non-racial outgroups, such as the homeless (Herrera et al., 2018), the colorblind (Ahn et al., 2013), and women (Lopez et al., 2019; Schulze et al., 2019). To the best of our knowledge, no study has examined the effect of embodiment in a co-ethnic immigration context in Asia, where virtual avatars are not differentiated by skin tone. As we are focused on ingroup-outgroup differences in an immigration context, the terms “Singaporean Chinese” and “PRC Chinese” will be used throughout the rest of this paper to distinguish between the two groups.

## Materials and Methods

### Participants

One-hundred-seventy-one undergraduate and graduate students from a university in Singapore participated in this experiment. Recruitment emails for participation were sent to a random selection of students from 15 student distribution email lists provided by the university, spanning across different faculties and courses. Participants (*N*=171) were all Singaporean Chinese, of whom 71 (41%) were men and 100 (58.5%) were women. The age range of participants spanned from 18 to 33 years old (*M*=22.43, *SD*=2.07). All students were compensated for participation with either course credit or SGD $10 gift cards.

### Experimental Design

The experiment featured four conditions in a 2 (embodiment order: PRC-first vs. Singaporean-first)×2 (number of embodiment scenarios: single-embodiment vs. double-embodiment) between-subjects design. The single-embodiment conditions, where participants only embodied one avatar, included *PRC*-*only* embodiment (i.e., PRC Chinese only; *n*=46) or *SG-only* embodiment (i.e., Singaporean Chinese avatar only; *n*=38). In the double-embodiment conditions, participants embodied two avatars sequentially in a counterbalanced order: the *PRC-then-SG* embodiment (i.e., PRC Chinese avatar first and Singaporean Chinese avatar second; *n*=41) and the *SG-then-PRC* embodiment (i.e., Singaporean Chinese avatar first then PRC Chinese avatar second; *n*=45).

In each embodiment scenario, participants were randomly assigned to embody either a food vendor or a drink vendor in a food court setting. In the PRC-then-SG and SG-then-PRC conditions, participants experienced both food and drink scenarios in a randomized counterbalanced order. In between the two scenarios, a transition scene informed participants which character they would embody next. A difference in character role was important to ensure participants did not undergo the same exact narrative, which could risk inducing maturation effects or boredom. Overall, the food and drink vendor scenarios had the same storyline and dialogue, with only changes in cosmetic details (i.e., the drink vendor prepared coffee, while the food vendor prepared noodles). As such, we did not count vendor role as a between-subjects factor in the design and analysis.

### Procedures

Upon arrival to the research laboratory, participants filled out an online consent form and pre-test questionnaire that measured their baseline perception and attitudes toward PRC Chinese immigrants in Singapore. They then were introduced to the HTC Vive headset and controls before entering a randomly assigned VR scenario. After the VR playthrough, they completed a post-test questionnaire, which included manipulation check questions and re-measured the dependent variables.

**Figure 1:**
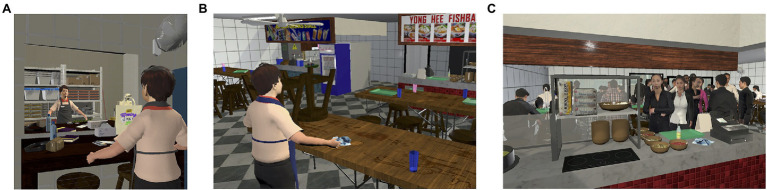
**(A)** In Scene 1, the participant begins by testing out their visuo-motor synchrony through a mirror in the storeroom. **(B)** In Scene 2, the participant interacts with the environment by setting up and cleaning the tables outside their stall. **(C)** In Scenes 3 and 4, the participant starts serving customers who enter the food court.

Through a pilot test with eight undergraduate participants, all stimuli used in the VR environment were developed and refined based on qualitative feedback. Within the VR environment, a voice narrator guided participants to complete tasks across four scenes (refer to Figure 1 for visuals). The participants’ character dialogue was voiced with a PRC Chinese accent and a Singaporean Chinese accent in the PRC embodiment and Singaporean embodiment scenarios, respectively. PRC Chinese and Singaporean Chinese student assistants were recruited to record voiceovers.

Scene 1: Participants were presented with a short introductory text pop-up explaining their embodied character’s role and background. Starting out in an ingredient storeroom, participants were made to observe their character’s appearance in a mirror and were prompted to test out their synchronized movements. They also interacted with their character’s personal belongings that emphasized their character’s identity. The character either had a Singaporean citizen’s identity card (pink in color) in the Singaporean Chinese scenario or a foreigner’s identity card (green in color) in the PRC Chinese scenario. Differentiating citizenship status by the color of official identity cards is a widely understood practice in Singapore (see Wan, 2015).

Scene 2: Outside of the storeroom, participants were first tasked with setting up the tables and chairs outside their respective stall. As the food vendor, they then had to replenish the supply of chili and spring onions in designated ingredient bowls. As the drink vendor, participants opened a can of evaporated milk and filled a kettle with hot water.

Scene 3: Participants started taking orders from virtual customers. In all conditions, participants then experienced a brief conflict with one Singaporean customer who expressed frustration as the participant could not understand the customer’s order. The customer is portrayed to be displeased in all conditions. In the PRC Chinese embodiment scenarios, the customer has an additional critique about the participant’s English proficiency. This addition was included to subtly highlight a realistic experience of discrimination faced by the PRC Chinese worker. The negative affect and narrative of this scene remained consistent for both PRC and Singaporean embodiment scenarios.

Scene 4: As participants are cleaning tables after customers have left, they received a phone call from their character’s child, who requested a considerable amount of money to be used as allowance for a school trip. At the conclusion of the scene, the narrator commented on how the character would need to find a way to provide for their child.

### Measures

The means, standard deviations, and Pearson’s correlations (*r*) of all measures are reported in Table 1. Only self-other overlap (i.e., closeness) and feeling thermometer scores (i.e., attitudes) were measured both pre-test and post-test. Difference scores were obtained by subtracting the mean pre-test rating from the mean post-test ratings for each of the two dependent variables. All other variables were measured only in the post-experiment questionnaire.

**Table 1:** Descriptive statistics and correlations between the feeling thermometer change scores, self-other overlap change scores, social identity orientation (SIO), and empathy.

S. No.		*M*	*SD*	1.	2.	3.	4.
1.	Δ Feeling thermometer	6.28	12.81	1			
2.	Δ Self-other overlap	0.42	0.96	0.297^**^	1		
3.	Social identity orientation	4.41	1.12	0.158^*^	0.183^*^	1	
4.	Empathy	5.01	1.45	0.306^**^	0.267^**^	0.228^**^	1

* *p<0.05;*

** *p<0.01*.

*M, mean; SD, standard deviation; Δ, change score*.

#### Self-Other Overlap (Closeness)

To measure perceived closeness, participants answered a single item modified and adapted from (Aron et al., 1992) questioning which of seven images best describes their relationship with PRC Chinese (1=no overlap at all, 7=high overlap). The prompt read: “Please select the picture below which best describes your relationship with a PRC Chinese.” Each picture featured two circles, each representing the “self” and “other.” From the first image to the last, the two circles progressively overlap with one another to represent greater degrees of self-other overlap. A higher score indicated greater perceived closeness between participants and PRC Chinese (pre-test: *M*=3.00, *SD*=1.41; post-test: *M=* 3.42, *SD=* 1.47).

#### Feeling Thermometer (Attitudes)

Attitudes toward outgroup members are measured by three items adapted from Alwin (1997). The prompt read: “Please indicate your attitudes toward PRC Chinese.” Items included “Cold (1) … Warm (100),” “Unfavourable (1) … Favourable (100),” and “Negative (1) … Positive (100).” A higher score indicated more positive attitudes (pre-test: *M*=57.41, *SD*=19.73, Cronbach’s *α=0*.92; post-test: *M=*63.69, *SD=* 18.84, Cronbach’s *α=0*.95).

#### Empathy

In the post-test questionnaire, participants were asked the extent to which they agreed or disagreed (1=strongly disagree, 7=strongly agree) with seven items, adapted from the Interpersonal Reactivity Index by Davis (1980), measuring their empathy levels toward PRC Chinese after the embodiment scenario. Items included “I felt as if I were in the shoes of the Chinese PRC” and “I felt compassion for the Chinese PRC.” A higher score indicated greater empathy toward PRC Chinese (*M=* 5.01, *SD=* 1.45, Cronbach’s *α=0*.95).

#### Social Identity Orientation

In the post-test questionnaire, participants completed 8 items adapted from Nario-Redmond et al. (2004) measuring the level of importance (1=not important at all, 7=extremely important) they ascribed to their social identity, with examples including “The memberships I have in various groups” and “My sense of belonging to my own racial group.” Note that the scale items are not designed to refer to a particular social identity, such as one’s Singaporean identity, but rather aim to get a general sense of participants’ propensity to identify at the social level more broadly. A higher score on this scale indicated a stronger SIO (*M*=4.41, *SD*=1.12, Cronbach’s *α*=0.84).

#### Manipulation Check

Participants were asked which character they represented in the VR context in the post-test questionnaire. Only participants in the double-embodiment conditions were made to indicate the correct order of embodiment. Participants in the PRC-only condition were significantly more likely than those in the SG-only condition to correctly report having represented a PRC Chinese character, *X*^2^(1, *N*=84)=80.05, *p*=0.000. Only one participant reported the incorrect character; as such, we did not exclude any data prior to analysis. All participants in the double-embodiment conditions reported the correct order of embodiment.

## Results

### Total Effect of Embodiment

To address the effects of embodiment on changes in feeling thermometer scores and self-other overlap, we first conducted a 2 (embodiment order)×2 (number of embodiment scenarios) between-subjects ANOVA with a Bonferroni adjustment for each dependent variable. Both tests ruled out the main effects of the number of embodiment scenarios and identified main effects of embodiment order, providing support for H2a.

Predicting changes in feeling thermometer attitudes, embodiment order had a significant main effect [*F*(1, 167)=6.54, *p*=0.011, $\eta_{p}^{2}$=0.04], such that embodying a PRC Chinese avatar first resulted in significantly more positive attitude change than embodying a Singaporean Chinese avatar first (*M*_diff_=4.91, *SE*=1.92, *p*=0.011). The main effect of the number of embodiment scenarios was not significant [*F*(1, 167)=1.60, *p*=0.207, $\eta_{p}^{2}$=0.01], nor was the interaction between embodiment order and the number of embodiment conditions [*F*(1, 167)=3.19, *p*=0.076, $\eta_{p}^{2}$=0.02].

A significant main effect of embodiment order likewise predicted changes in self-other overlap [*F*(1, 167)=4.52, *p*=0.035, $\eta_{p}^{2}$=0.03], such that embodying a PRC Chinese avatar first led to significantly greater self-other overlap than embodying a Singaporean Chinese avatar first (*M*_diff_=0.31, *SE*=0.15, *p*=0.035). Neither the main effect of the number of embodiment scenarios [*F*(1, 167)=0.01, *p*=0.924, $\eta_{p}^{2}$=0.00], nor its interaction with embodiment order [*F*(1, 167)=3.73, *p*=0.055, $\eta_{p}^{2}$=0.02] was significant.

**Figure 2:**
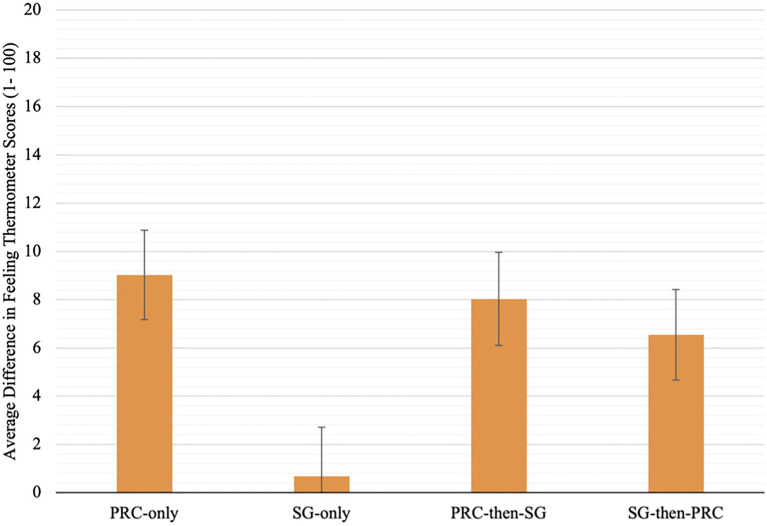
Mean pre-to-post-change in feeling thermometer scores (1–100) for each embodiment condition. Error bars represent standard errors of the mean.

Having discounted the two-way interaction and a confounding effect of the number of embodiment conditions, we then conducted two separate one-way ANOVAs to assess multiple contrasts between the four embodiment conditions, with the SG-only condition set as a control. For feeling thermometer difference scores, the ANOVA model was significant, [*F*(3, 170)=3.54, *p*=0.016, $\eta_{p}^{2}$=0.06]. *Post hoc* tests with a Tukey HSD adjustment indicate that significant differences only exist between the PRC-only and SG-only groups, and between the PRC-then-SG and SG only groups. Participants in the SG-only condition experienced significantly less change in attitudes toward the PRC Chinese than those in the PRC-only condition (*M*_diff_=−8.34, *SE*=2.75, *p*=0.015) and the PRC-then-SG only condition (*M*_diff_=−7.35, *SE*=2.81, *p*=0.047). These results provide support for H1. Contrary to H2a, despite a main effect found for the PRC-first embodiment factor, no significant differences were found between the SG-then-PRC and PRC conditions, nor between the SG-then-PRC and PRC-then-SG conditions (*p*s>0.05; see Figure 2 for mean differences). However, supporting H2b, there was no significant difference between the SG-then-PRC and SG-only conditions.

**Figure 3:**
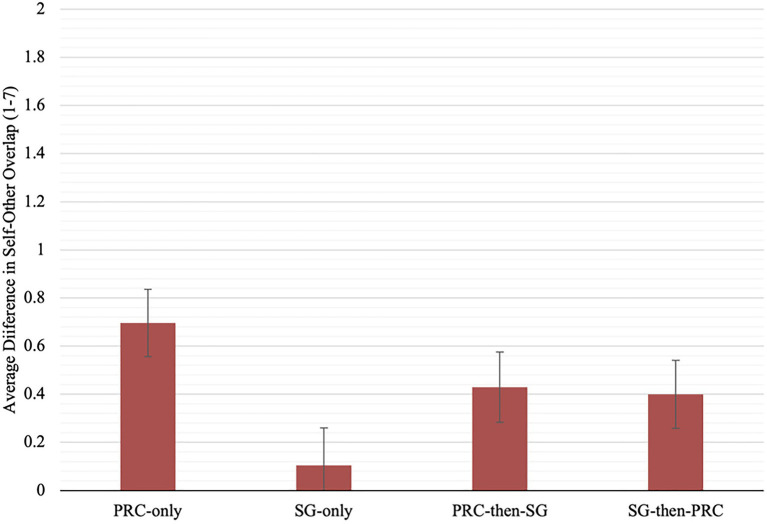
Mean pre-to-post-change in self-other overlap (1–7) for each embodiment condition. Error bars represent standard errors of the mean.

A significant effect of embodiment was also found on difference scores in self-other overlap, [*F*(3, 170)=2.70, *p*=0.048, $\eta_{p}^{2}$=0.05]. As the assumption of homogeneity of variances was violated in this analysis, a Games–Howell *post hoc* adjustment was utilized to deduce group differences. Participants in the SG-only embodiment condition displayed significantly less change in overlap with PRC Chinese than those who underwent PRC-only embodiment (*M*_diff_=−0.59, *SE*=0.18, *p*=0.010), partially supporting H1. Difference scores in the SG-then-PRC group did not significantly contrast with scores in the PRC-then-SG, PRC-only and SG-only conditions (*p*s>0.05), likewise supporting H2b but contradicting H2a (see Figure 3 for mean differences).

### Indirect Effect of Embodiment *via* Empathy

To explore mediation effects, we utilized the method recommended by Hayes and Preacher (2013) for mediation with a multi-categorical antecedent. This approach involves conducting multiple ordinary least squared (OLS) regression models that deduce the relative indirect effects of each condition *via* empathy against the control, while the effects of the other manipulation groups are held constant (Hayes and Preacher, 2013). To assess the relative indirect effects of the PRC-only, PRC-then-SG, and SG-then-PRC conditions against the SG-only group, we ran Model 4 with 10,000 bootstraps in the PROCESS macro developed by Hayes (2012) for SPSS v24. Z-scores were computed for all continuous variables to generate standardized path estimates.

#### Feeling Thermometer

**Figure 4:**
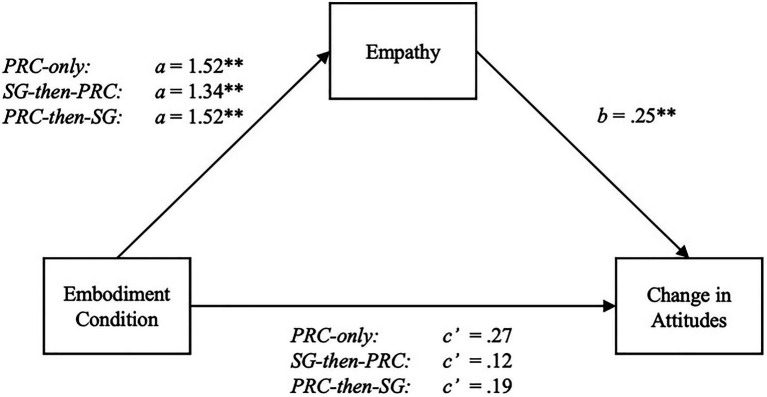
Statistical model of empathy mediating the relative effects of embodiment condition on differences in feeling thermometer scores (attitudes). Coefficients are based on z-scored standardized values for empathy and feeling thermometer change scores. ^*^p<0.05; ^**^p<0.01.

The model predicting standardized feeling thermometer difference scores was significant with the dummy-coded PRC-only, PRC-then-SG, and SG-then-PRC conditions, as well as empathy, set as predictors [*F*(4, 166)=4.61, *R*^2^=0.10, *MSE*=0.92, *p*=0.002]. An omnibus test revealed a significant total direct effect of embodiment condition on feeling thermometer differences [*F*(3, 167)=3.54, *R*^2^=0.06, *p*=0.016]. Separately, the relative total effects of the PRC-only [*β*=0.65, *t*(82)=3.04, *p*=0.003 (0.23, 1.07)], SG-then-PRC [*β*=0.46, *t*(81)=2.12, *p*=0.035 (0.03, 0.88)], and PRC-then-SG conditions [*β*=0.57, *t*(78)=2.62, *p*=0.010 (0.14, 1.01)] were significant. Notably, none of the relative direct effects of embodiment were significant [*F*(3, 166)=0.41, *R*^2^=0.01, *p*=0.75]. The relative indirect effects, however, were all significant, as the omnibus indirect effect confidence interval did not include zero [*β*=0.09, *SE*=0.04 (0.03, 0.18)]. The effects of the PRC-only [*β*=0.39, *SE*=0.15 (0.14, 0.73)], SG-then-PRC [*β*=0.34, *SE*=0.13 (0.13, 0.65)], and PRC-then-SG conditions [*β*=0.39, *SE*=0.15 (0.14, 0.72)] were positively mediated by empathy. The presence of indirect effects and the absence of direct effects support H3 (see Figure 4).

#### Self-Other Overlap

**Figure 5:**
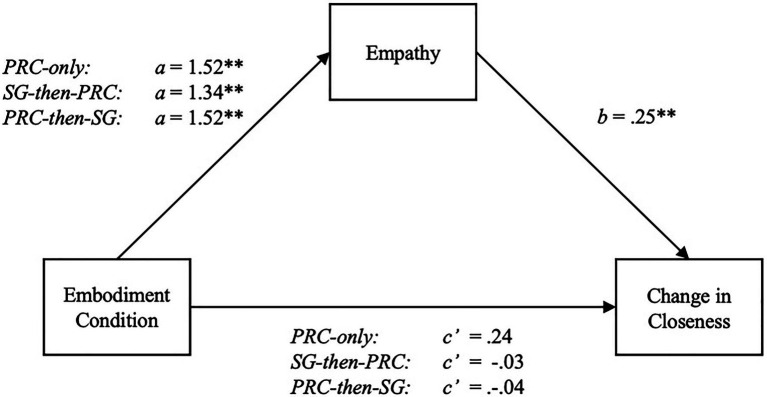
Statistical model of empathy mediating the relative effects of embodiment condition on differences in self-other overlap (closeness). Coefficients are based on z-scored standardized values for empathy and self-other overlap change scores. ^*^p<0.05; ^**^p<0.01.

With the dummy-coded PRC-only, PRC-then-SG, and SG-then-PRC conditions and empathy set as predictors for standardized differences in self-other overlap, the overall model was significant [*F*(4, 166)=3.83, *R*^2^=0.08, *MSE*=0.94, *p*=0.005]. The overall total effect of embodiment condition on difference scores was significant [*F*(3, 167)=2.69, *R*^2^=0.05, *p*=0.048]. The PRC-only condition was the only one that had a significant, relative total effect on self-overlap differences compared to the SG-only group [*β*=0.61, *t*(82)=2.84, *p*=0.005 (0.19, 1.04)]. Nonetheless, indirect effects can still exist in the apparent absence of a total effect, as the statistical power to detect an indirect effect may exceed the power used to determine a total effect. These indirect effects are thus still worth investigating (Rucker et al., 2011; Hayes, 2017). True enough, the relative indirect effect of embodiment condition *via* empathy was found to be significant, as the confidence interval for the omnibus effect did not include zero [*β*=0.09, *SE*=0.03 (0.03, 0.17)]. Analyzed separately, the PRC-only [*β*=0.38, *SE*=0.14 (0.14, 0.69)], SG-then-PRC [*β*=0.33, *SE*=0.13 (0.12, 0.63)], and the PRC-then-SG [*β*=0.38, *SE*=0.14 (0.13, 0.70)] all had significant positive indirect effects on self-other overlap differences through empathy. None of the relative direct effects were significant, providing support for H3 (see Figure 5).

### Moderated Mediation With Social Identity Orientation

Higher aggregate scores for SIO positively predicted greater improvements in feeling thermometer scores [*b*=1.81, *β*=0.158, *t*(170)=2.08, *p*=0.04] and self-other overlap [*b*=0.157, *β*=0.183, *t*(170)=2.42, *p*=0.02]. In line with the past research, there was also a significant positive relationship between SIO and empathy [*b*=0.29, *β*=0.23, *t*(170)=3.04, *p*=0.003]. Given the significant relationship between SIO and outcome variables, we tested the possibility of SIO moderating the both the direct and indirect of embodiment condition *via* empathy on changes in both self-other overlap and feeling thermometer scores. We utilized Model 15 in the PROCESS Macro with 10,000 bootstraps and standardized all continuous variables.

#### Feeling Thermometer

The overall model predicting feeling thermometer differences was significant [*F*(9, 161)=2.56, *R*^2^=0.13, *p*=0.009]. However, the indices of moderated mediation with PRC-only [*β*=0.07, *SE*=0.12 (−0.14, 0.35)], PRC-then-SG [*β*=0.06, *SE*=0.11 (−0.13, 0.31)], and SG-then-PRC [*β*=0.07, *SE*=0.12 (−0.15, 0.35)] as focal predictors were not significant, as the bootstrapped confidence intervals all included zero. As such, there was no evidence to suggest that SIO moderated the effect of embodiment condition on attitude change *via* empathy.

#### Self-Other Overlap

**Figure 6:**
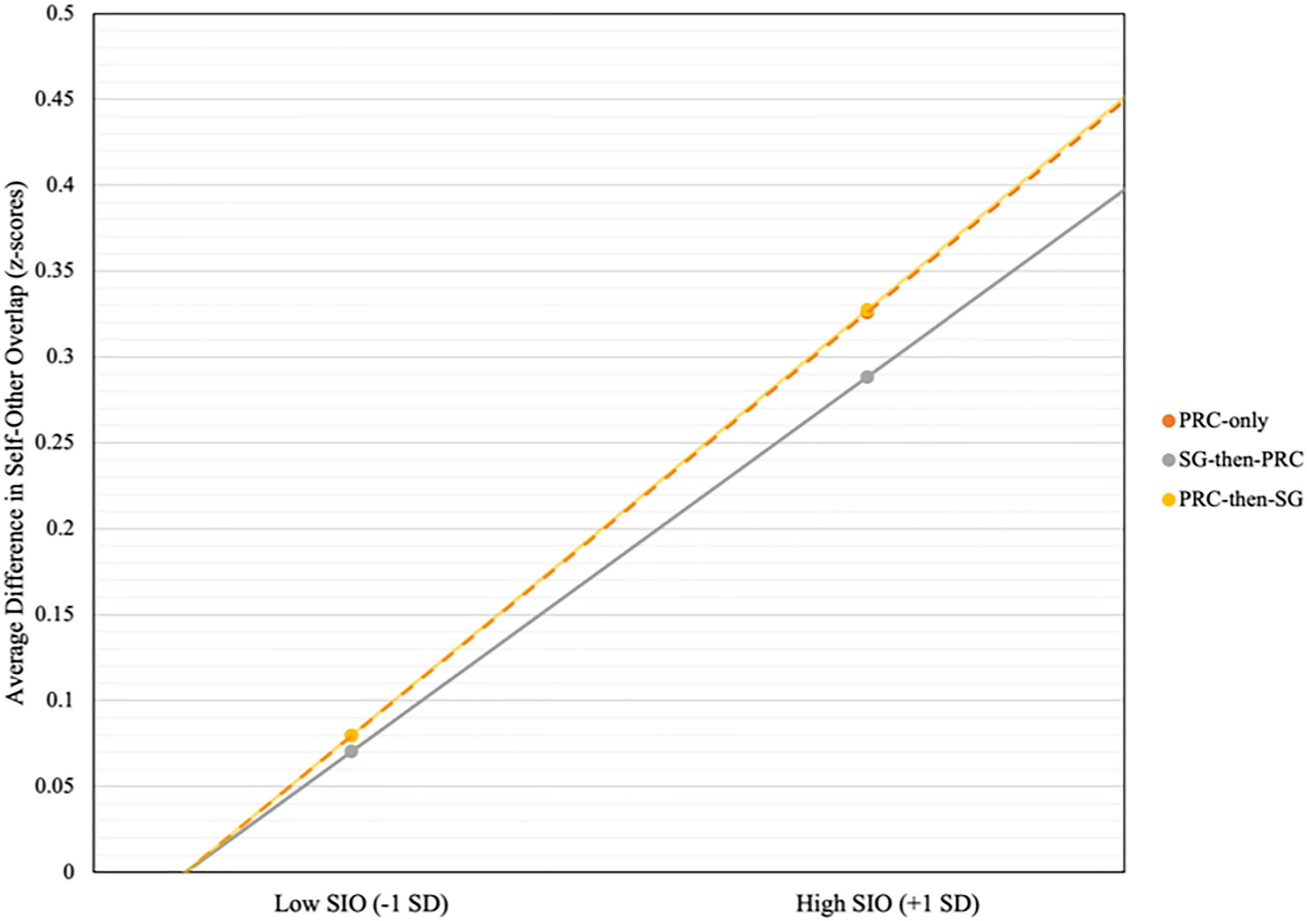
Comparison of indirect effects of the PRC-only, SG-then-PRC, and PRC-then-SG conditions on differences in self-other overlap (closeness) moderated by social identity orientation (SIO). Slopes represent conditional indirect effects of each embodiment condition *via* empathy, based on values when SIO is −1 SD and+1 SD from the mean. For high SIO (+1 SD) participants, all three conditions produced significant indirect effects.

The overall model predicting self-other overlap difference scores was significant [*F*(9, 161)=3.28, *R*^2^=0.15, *MSE*=0.83, *p*=0.001]. Furthermore, the indices of moderated mediation with PRC-only [*β*=0.25, *SE*=0.12 (0.01, 0.50)], PRC-then-SG [*β*=0.22, *SE*=0.11 (0.01, 0.45)] and SG-then-PRC [*β*=0.25, *SE*=0.13 (0.02, 0.52)] as focal predictors were all significant, as the bootstrapped confidence intervals did not include zero. Empathy positively predicted changes in self-other overlap when SIO was high [+1 SD; *β*=0.38, *SE*=0.13, *p*=0.004 (0.13, 0.63)], but not low [−1 SD; *β*=0.05, *SE*=0.13, *p*=0.69 (−0.20, 0.31)]. Further probing into these conditional effects, we found that only the relative indirect effects of each PRC embodiment condition varied by SIO. When SIO was high (+1 SD), the three conditions significantly improved self-other overlap (compared against the SG-only control) indirectly *via* empathy but not directly. In contrast, when SIO was low (−1 SD), the effect of PRC-embodiment was not significant, whether direct or indirect (see Figure 6). Conditional effects of each condition are presented in Table 2.

**Table 2:** Direct and indirect effects *via* empathy of PRC embodiment on change in self-other overlap, conditional on social identity orientation (SIO). Values are based on z-scored empathy, SIO, and change in self-other overlap.

		Direct effect			Indirect effect		
Condition	SIO values	*β*	*SE*	*CI*	*β*	*SE*	*CI*
PRC-only	+1 SD	0.50	0.37	−0.22, 1.23	0.57^*^	0.21	0.18, 1.00
	−1 SD	0.13	0.36	−0.58, 0.83	0.08	0.15	−0.24, 0.38
PRC-then-SG	+1 SD	−0.04	0.34	−0.70, 0.63	0.51^*^	0.19	0.15, 0.90
	−1 SD	0.05	0.36	−0.66, 0.76	0.07	0.14	−0.21, 0.35
SG-then-PRC	+1 SD	−0.31	0.36	−1.02, 0.40	0.58^*^	0.21	0.17, 1.02
	−1 SD	0.28	0.33	−0.38, 0.94	0.08	0.16	−0.24, 0.38

* *CI does not include zero*.

*β, standardized effect; SE, standard error; CI, confidence interval*.

There was a marginally significant interaction between empathy and SIO, [*β*=0.11, *t*(170)=0.1.87, *p*=0.064 (−0.01, 0.33)]. In contrast, the highest-order interaction between embodiment condition and SIO was not significant [*β*=0.18, *t*(82)=0.73, *p*=0.47 (−0.32, 0.70)]. Though significant interaction coefficients are neither required nor sufficient to determine an overall moderated mediation effect (Hayes, 2015), the clear lack of a significant embodiment by SIO interaction does align with the absence of conditional direct effects.

Accounting for the possibility of a first-stage moderated mediation in the model, we tested whether embodiment condition interacted with SIO to predict empathy. The interactions were not significant with PRC-only [*β*=−0.01, *t*(82)=−0.03, *p*=0.97 (−0.36, 0.35)], PRC-then-SG [*β*=−0.11, *t*(82)=−0.63, *p*=0.53 (−0.44, 0.23)], and SG-then-PRC groups [*β*=0.08, *t*(82)=0.53, *p*=0.59 (−0.23, 0.40)] as focal predictors. As such, there was no evidence to suggest conditional effects of embodiment on empathy.

**Figure 7:**
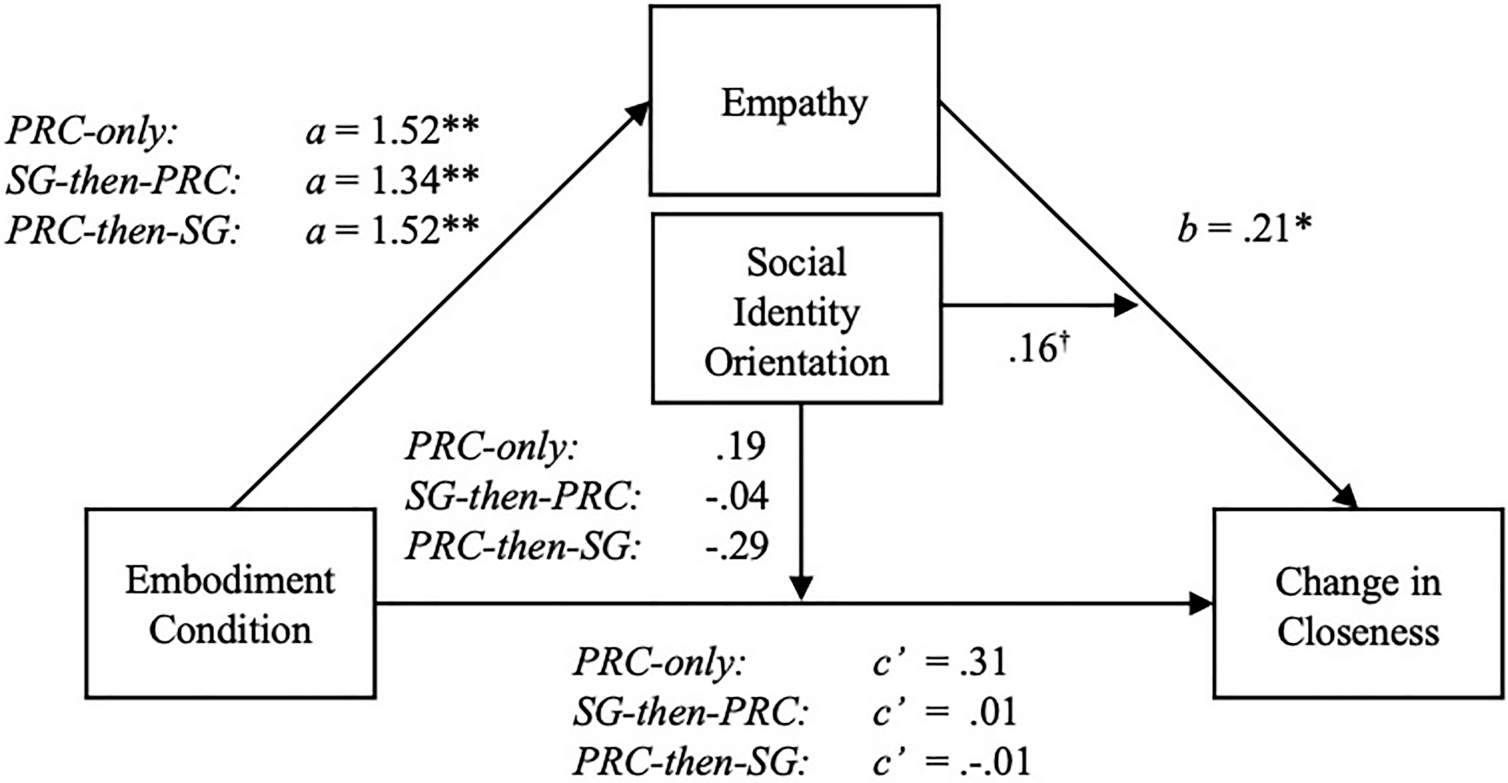
Statistical moderated mediation model with SIO moderating the second-stage indirect effects of embodiment *via* empathy on changes in self-other overlap. Coefficients are based on z-scored standardized values for empathy, SIO, and self-other overlap change scores. ^*^p<0.05; ^**^p<0.01; ^†^*p*<0.07.

The absence of conditional direct effects suggests that in response to RQ3, SIO moderates only the indirect effects of embodiment *via* empathy, not the direct effects (see Figure 7).

Findings from all analyses are summarized in Table 3.

**Table 3:** Summary of main analyses and significant effects. Cells indicate embodiment conditions that resulted in significant effects (contrasted with the SG-only condition) for each analysis.

DV Effect	Feeling thermometer scores (attitudes)	Self-other overlap (closeness)
Total effect of embodiment (one-way ANOVA)	PRC-only PRC-then-SG	PRC-only
Indirect effect *via* empathy (mediation)	PRC-only PRC-then-SG SG-then-PRC^*^	PRC-only PRC-then-SG^*^ SG-then-PRC^*^
Moderation of indirect effect *via* empathy by social identity orientation (moderated mediation)		PRC-only PRC-then-SG^*^ SG-then-PRC^*^

* *Significant indirect effects were found (*i.e.*, confidence interval does not include zero) despite the absence of significant total effects in the one-way ANOVAs*.

## Discussion

### The Effect of Outgroup Embodiment

Findings from this study support past research on how outgroup embodiment can result in more positive attitudes toward a salient outgroup (Peck et al., 2013; Banakou et al., 2016; Hasler et al., 2017). Mirroring research on perspective-taking (Galinsky et al., 2005), participants exposed to the outgroup-only embodiment scenario also reported significantly greater gains in self-other overlap with the outgroup than participants in the ingroup-only control. Whereas past embodiment research focused on assessing attitudes (e.g., Groom et al., 2009; Peck et al., 2013) and prosocial intentions (Herrera et al., 2018), our study additionally demonstrates that effects can be induced for cognitive perceptions of self-outgroup merging as well.

Two of three outgroup embodiment conditions were successful in improving attitudes relative to the ingroup condition. However, while both the outgroup-only and outgroup-then-ingroup conditions were found to have significant total effects on attitude change, only the outgroup-only condition produced a significant total effect on self-other overlap. Conceptually, while the feeling thermometer scale is utilized as a direct measure of attitudes self-other overlap measures perceptions of the self in relation to the other (Aron et al., 1992; Galinsky et al., 2005). The outgroup-then-ingroup manipulation involved both self- *and* other-embodiment, potentially distracting participants from concentrating fully on the “other” perspective. Moreover, by embodying both identities, participants’ attention may have inadvertently been drawn to differences in ingroup and outgroup experiences instead of the similarities or overlap between groups.

In contrast to the majority of studies testing the effect of outgroup embodiment in a racial context, our study demonstrates improved intergroup attitudes in a co-ethnic context, where there are no visually salient distinctions by skin tone. Outgroups perceived as too similar to an ingroup may threaten the uniqueness or novelty of the ingroup’s social identity, thus resulting in negative attitudes as a defensive mechanism (Binggeli et al., 2014). Thus, while individuals may identify with groups to bolster their self-concept, maintaining some level of distinctiveness is still crucial (Hornsey and Hogg, 2000). Our Singaporean Chinese participants may be especially reluctant to associate themselves with the broad “Chinese” label they share with PRC Chinese, as doing so may risk sacrificing their distinct sub-group cultural identity. Our study contributes to the very scarce body of research on mitigating intraethnic divides (Clark, 2004), where the desire for positive distinctiveness may be especially heightened due to superficial group similarities.

### Anchoring and Order Effects

The order of perspective-taking *via* embodiment matters, albeit to a limited extent. Supporting H2b, no differences were found between the ingroup-then-outgroup and the ingroup-only conditions, despite the former including an outgroup embodiment scenario. In the ingroup-then-outgroup condition, undergoing the ingroup scenario condition first may have enhanced the salience and accessibility of the ingroup perspective as an anchor (Mussweiler and Strack, 1999a), thus increasing the amount of cognitive resources required for participants to adjust to the subsequent outgroup perspective (Epley et al., 2004). These findings thus support the literature on how the adjustment required in perspective-taking can be limited in effectiveness due to the stability of an egocentric anchor (Mussweiler and Strack, 1999a,b).

H2a was partially supported. We found a main effect of order on attitudes and closeness, such that outgroup-first conditions generated more positive shifts in bias than ingroup-first conditions. These findings provide support for a primacy effect when evaluating contrasting pieces of evidence in simple judgment tasks (Hogarth and Einhorn, 1992). Through a subsequent contrast analysis, we also found that the outgroup-then-ingroup condition significantly influenced changes in attitudes relative to the ingroup-only control group, while the ingroup-then-outgroup condition did not.

However, contrary to predictions, there were no significant differences between the ingroup-then-outgroup and the outgroup-only/outgroup-then-ingroup conditions, where outgroup embodiment was induced first or alone. In a preliminary analysis, we found that within the ingroup-then-outgroup condition, both attitudes and perceived closeness significantly improved from pre-test to post-test, whereas no improvements were found in the ingroup-only group (refer to Supplementary Material). It is possible that that anchoring effects worked in opposition to latent effects of embodiment in the ingroup-then-outgroup condition, both sides “cancelling” each other out to an extent. Due to competing influences, the attitudinal change generated by this manipulation may have not been different enough from other conditions to generate statistical significance. While anchor-consistent information can strengthen an anchor, anchor-inconsistent information can destabilize the anchor (Mussweiler et al., 2000; Strack et al., 2016). In the ingroup-then-outgroup condition, perhaps the immersive outgroup embodiment scenario helped to destabilize the anchor induced by the ingroup embodiment scenario, although not completely.

Additive explanations to anchoring, such as mental fatigue, are plausible. Extended exposure and repeating similar tasks during VR may hinder participants’ performance due to an onset of visual and cognitive fatigue (Malińska et al., 2015). We designed two different scenarios (food and drink vendors) to prevent repetitiveness, but the narrative and dialogue remained mostly the same. As such, participants in the double-embodiment conditions had to undergo similar scenes twice, potentially contributing to fatigue. This fatigue may have added on to the cognitive load induced by anchoring effects in the ingroup-then-outgroup condition, hindering perspective-taking efforts. When tired, people tend to form quick and easy judgments without excessive thought to conclude an activity (Webster et al., 1996).

### Empathy as a Mediator

A notable finding from the present study is the role of empathy as a complete mediator of the effect of embodiment on attitudes and self-other overlap of an outgroup. These results support the few studies that identified empathy as a partial mediator between perspective-taking and intergroup attitudes (Vescio et al., 2003; Shih et al., 2009). Embodiment may be a robust mechanism for inducing empathy toward an outgroup. In fact, significant indirect effects of empathy on attitude change were present for all three outgroup manipulation conditions, despite the absence of total effects. This outcome can be explained by how the relationship between outgroup embodiment and empathy was stronger than the overall effect of outgroup embodiment on the dependent variables (Rucker et al., 2011).

Nonetheless, Rucker et al. (2011) caution against describing a statistical mediator as “full” or “complete,” given that any regression model does not account for all possible mediators and moderators at the same time. One possibility for the absence of total effects may lie in suppression effects (MacKinnon et al., 2000), where our model may have not accounted for other variables that mediated the effect of outgroup embodiment in the opposite direction (Rucker et al., 2011). We qualify that empathy may only act as a “full” mediator of the observed effect of embodiment given the scope of our study and variables of interest. Further research should directly compare the indirect effect sizes of empathy (as a mediator) in VR embodiment versus imaginal perspective-taking manipulations and account for other mediators that could explain variance in this effect. The present study is the first, to our knowledge, to situate VR embodiment, empathy, and intergroup attitudes in a path analysis.

### Social Identity Orientation as a Moderator

The effect on self-other overlap was moderated by participants’ level of importance ascribed to social identity. These findings contribute to the divisive literature on how identity dispositions influence negative intergroup attitudes and are the first to test this construct in relation to embodiment. The stronger one’s SIO is, the more effective embodiment is in improving self-other overlap with the outgroup. These findings provide additional evidence for the attentional hypothesis proposed by Wu and Keysar (2007). People with a SIO define the self in relational terms and thus perceive the role of others’ behavior and thoughts to be more significant (Markus and Kitayama, 1991). They may be less prone to egocentrism and are more receptive to identifying commonalities with others (Wu and Keysar, 2007).

Through a moderated mediation analysis, we also found that this moderation solely impacts the indirect effect of outgroup embodiment *via* empathy. Findings thus extend past literature on how related constructs such as collectivism (Duan et al., 2008), interdependence (Cross et al., 2000; Chen et al., 2020), and social group awareness (Zhao et al., 2013) are conducive to empathic expression. Attaching importance to social identity may heighten one’s propensity toward forging bonds and solidarity with others, thus enabling the prosocial benefits of empathy to manifest.

SIO notably did not moderate the first-stage relationship between embodiment and empathy. It is possible that the *type*, rather than the existence of empathy, depends on SIO. Research has found that collectivists, who value the social collective, are more likely to develop emotional empathy (i.e., the vicarious experience of others’ emotions), while individualists, who value personal distinctiveness, are drawn to cognitive empathy (i.e., the mental understanding of another’s perspective; Duan et al., 2008). A stronger SIO may then relate to emotional empathy development, where participants feel the pain and struggles of the PRC Chinese first-hand. Being this emotionally involved may encourage feelings of intimacy and closeness with the other (Roberts et al., 2014), possibly to a greater extent than cognitive empathy, which is less personally involving and taxing (Duan, 2000). More research should be done to unpack how dimensions of empathy differentially influence prosocial outcomes, preferably with a more robust sample size for this analysis.

However, SIO did not significantly moderate the effect of embodiment on attitudinal change, despite trending in the expected direction. The difference may lie in the stronger conceptual connections between identity orientations and self-other overlap, which both focus on the cognitive self-concept. Those with a SIO are more attuned to self-identity *in relation* to others (Nario-Redmond et al., 2004)—in turn, this disposition may make one more receptive to merging the ‘self’ with the ‘other.’

### Limitations

The study is not without its limitations. In our test of order effects, the double-embodiment manipulation conditions inevitably required twice as much time than the single-embodiment conditions to complete. Though this concern was mitigated by a nonsignificant main effect of the number of embodiment conditions, it is unclear whether time or effort may have influenced results in unidentified ways. Furthermore, SIO was also analyzed as a quasi-independent variable and not manipulated, so although moderation findings are predictive, direct causality cannot be inferred. Due to time and resource constraints, we also did not gauge implicit attitudes as a measure of intergroup attitudes (e.g., Groom et al., 2009; Peck et al., 2013; Lopez et al., 2019). It is possible that our dependent measures were limited in fully capturing non-conscious thoughts and avoiding demand characteristics.

As we strove to achieve ecological validity and realism in the embodiment scenario, we included an additional customer critique about English proficiency toward the PRC Chinese migrant avatar in the PRC embodiment scenario. Outside of this addition, the ingroup and outgroup embodiment scenarios were kept identical in terms of dialogue and storyboard. However, we acknowledge that the slightly more explicit negative feedback conveyed to the outgroup avatar may have inflated the apparent effects of embodiment on empathy for the outgroup. Future research should empirically test how different levels of positive versus negative experiences in outgroup (versus ingroup) embodiment can influence intergroup outcomes.

## Conclusion

The present study addresses the research gap regarding the psychological processes underlying VR avatar embodiment. It extends scarce research on the role of trait variables, affective responses, and cognitive heuristics that may serve as underlying mechanisms or boundary conditions for embodiment to work effectively. Findings showed that virtual embodiment of an outgroup avatar significantly improved attitudes and self-other overlap with the outgroup. Furthermore, the study demonstrated this effect in a novel co-ethnic immigration context. Extending embodiment research, we find that the effects of embodiment on intergroup bias are strongly mediated by empathy. However, the prosocial benefits of this manipulation may also be contingent on the strength of one’s SIO. The order of embodiment also matters to a certain extent due to potential anchoring effects. This study has important implications for the application of VR avatar embodiment to intergroup tensions. Moving forward, future research should further delve into these mechanisms to optimize virtual embodiment strategies for various contexts.

## Data Availability Statement

The datasets presented in this article are not readily available because the data is under restriction of the Data Management Plan (DMP) approved by Nanayang Technological University and Ministry of Education Singapore (funder). Requests to access the datasets should be directed to VC, the corresponding author, at chenhh@ntu.edu.sg.

## Ethics Statement

The studies involving human participants were reviewed and approved by Institutional Review Board, Nanyang Technological University, Singapore. The patients/participants provided their written informed consent to participate in this study.

## Author Contributions

VC contributed to the overall conceptualization of the study, study design, operationalization of variables, the virtual reality environment design, writing of the paper, as well as supervised the data collection and data analysis. GI contributed to the literature search, literature review, formation of arguments, and writing of all sections of the manuscript and conducted data analysis. VL and JL contributed to the literature search, a preliminary summary of the literature, operationalization of the variables, and VR design ideas and conducted experiment sessions. All authors contributed to the article and approved the submitted version.

## Funding

This research is supported by the Ministry of Education, Singapore, under its Academic Research Fund Tier 2 Grant (MOE2017-T2-2-145).

## Conflict of Interest

The authors declare that the research was conducted in the absence of any commercial or financial relationships that could be construed as a potential conflict of interest.

## Publisher’s Note

All claims expressed in this article are solely those of the authors and do not necessarily represent those of their affiliated organizations, or those of the publisher, the editors and the reviewers. Any product that may be evaluated in this article, or claim that may be made by its manufacturer, is not guaranteed or endorsed by the publisher.
