# Supplementary material for: The Effect of VR Avatar Embodiment on Improving Attitudes and Closeness Toward Immigrants
Source: Front Psychol. 2021 Oct 15;12:705574. doi: 10.3389/fpsyg.2021.705574 (PMC8554103; doi:10.3389/fpsyg.2021.705574)
Supplement: Supplementary file 1 [file Data_Sheet_1.docx]

Supplementary Material

# Analysis of Pre-Post Changes in Attitudes and Closeness per Condition

As a preliminary analysis, a series of paired sample t-tests were conducted separately for each of the four embodiment conditions (PRC-only, SG-only, PRC-then-SG, and SG-then-PRC), assessing mean differences in pre-test and post-test scores for feeling thermometer scores and self-other overlap. As our primary hypotheses are concerned with comparing the change in dependent variables (i.e., difference scores) between conditions, this within-subjects analysis was excluded from the main manuscript. These findings, nonetheless, provide preliminary insight into the effectiveness of the PRC-only, PRC-then-SG, and SG-then-PRC manipulations in stimulating a change in attitudes and closeness toward PRC Chinese. See Figure 1 and Figure 2 for pre-post comparisons of feeling thermometer scores and self-other overlap, respectively.

For participants in the PRC-only condition, pre-test and post-test scores were significantly different across both feeling thermometer scores [*t*(45) = 3.84, *p* = .000] and self-other overlap [*t*(45) = .70, *p* = .000]. After going through the study, participants reported higher feeling thermometer scores, [*M* _diff_  **=** 9.03, *SE* = 2.35] and greater self-other overlap with PRC Chinese [*M* _diff_  **=** .67, *SE* = .16].

Similar to the PRC-only condition, participants in the PRC-then-SG condition exhibited significant differences across the dependent variables in favor of PRC Chinese, including greater feeling thermometer scores [*t*(41) = 4.45, *p* = .000, *M* _diff_  **=** 8.03, *SE* = 1.80] and greater self-other overlap [*t*(41) = 2.61, *p* = .012, *M* _diff_  **=** .43, *SE* = .16].

Likewise, participants in the SG-then-PRC condition also reported higher scores on the feeling thermometer scale [*t*(44) = 3.55, *p* = .001, *M* _diff_  **=** 6.55, *SE* = 1.85] and greater self-other overlap with PRC Chinese [*t*(44) = 2.79, *p* = .008, *M* _diff_  **=** .40, *SE* = .14].

In contrast to the three aforementioned conditions with PRC-embodiment scenarios, the SG-only condition did not generate any significant pre-post differences in either feeling thermometer scores [*t*(37) = .52, *p* = .608, *M* _diff_ *=* .68, *SE* = 1.32] or self-other overlap with the PRC Chinese [*t*(37) = 1.16, *p* = .254, *M* _diff_ *=* .11, *SE* = .09].

**Figure 1.** Mean averages between pre-test and post-test feeling thermometer scores are compared for each manipulation condition. Error bars represent standard errors of the mean. *Difference between means is significant at the *p* < 0.01 level.

**Figure 2.** Mean averages between pre-test and post-test feeling thermometer scores are compared for each manipulation condition. Error bars represent standard errors of the mean. *Difference between means is significant at the *p* < 0.05 level. ** Difference between means is significant at the *p* < 0.01 level.
